# Dysregulation of the hypothalamic-pituitary-adrenal system in Tuvan alcoholics is associated with a high level of stress in comparison with ethnic Russian patients

**DOI:** 10.1192/j.eurpsy.2022.1395

**Published:** 2022-09-01

**Authors:** T. Shushpanova, N. Bokhan, A. Mandel, T. Novozheeva

**Affiliations:** 1 Mental Health Research Institute of Tomsk National Investigation MedicalCenter of Russian Academy of Sciences, Clinical Psychoneuroimmunology And Neurobiology, Tomsk, Russian Federation; 2 Siberian State Medical University, Department Of Psychiatry, Narcology And Psychotherapy,, Tomsk, Russian Federation; 3 Mental Health Research Institute Tomsk National Research Medical Center Russia Academy of Science, , Department Of Addictive States, Tomsk, Russian Federation

## Abstract

**Introduction:**

Alcoholization is considered as prolonged auto-aggression, low frustration tolerance, filling of the existential vacuum. In all cases, the use of a psychoactive substance that alters the state of consciousness is regarded as a way of escape from real life.

**Objectives:**

The study of clinically significant markers in alcoholism associated with the constitutional characteristics of craving for alcohol in people of different ethnicity is importance for the formation of new approaches to prevention and treatment.

**Methods:**

68 Russian alcoholics and 67 Tuvans alcoholics only men and 20 healthy male were monitored. Clinical assessment of the condition of patients was carried out with the traditional clinical description. Enzyme-linked immunosorbent assay kits were used to determine serum hormone levels in patients and volunteers.

**Results:**

Deeper shifts in the increase in ACTH and Cortisol levels were found in alcoholic Tuvinians compared with Russian patients, which is associated with a high risk of alcohol dependence and a highly progressive course of the disease. The index of the ratio Cortisol/ACTH (IR) in the blood of alcoholic patients of the Russian (IR - 10,36) and Tuvan (IR - 10,62) nationalities does not differ, but significantly (1.5 times) differs from the indicator in healthy individuals (IR - 15,12).

**Conclusions:**

The background level of dysregulation of the hypothalamic-pituitary-adrenal axis in patients of Tuvan nationality is significantly more pronounced, however, the index of Cortisol/ACTH ratios (IR) in each ethnic group of patients is constant in this disease, which is characterized by a high level of stress.

**Disclosure:**

No significant relationships.

